# The role of attitude toward nature in learning about environmental issues

**DOI:** 10.3389/fpsyg.2024.1471026

**Published:** 2024-11-13

**Authors:** Tessa-Marie Baierl, Florian G. Kaiser, Franz X. Bogner

**Affiliations:** ^1^Department of Biology and Chemistry Education, University of Bayreuth, Bayreuth, Germany; ^2^Institute of Psychology, Otto-von-Guericke University Magdeburg, Magdeburg, Germany; ^3^Earth Education Research and Evaluation Team, College of Education, University of Arizona, Tucson, AZ, United States

**Keywords:** attitude toward nature, environmental attitude, environmental education, attitude measurement, adolescents, Campbell paradigm

## Abstract

Attitude toward nature and environmental attitude are two distinct propensities that both further learning about the environment. The present study builds upon prior research by investigating the role of attitude toward nature in learning about environmental issues. In a sample of 1,486 university, middle and high school students (*M_age_* = 15.25, *SD* = 3.2), we first calibrated a pool of items expressing attitude toward nature. We found differences in how adolescents expressed their appreciation for nature at different ages. It is essential to consider these differences to accurately ascertain adolescents’ attitudes toward nature. We then conducted a mediation test. Whereas attitude toward nature determined the levels of knowledge students gained and retained, environmental attitude fully mediated the environmental knowledge subsequently demonstrated by the students. Our research suggests that researchers and educators may benefit from taking an experiential approach to learning about sustainable development by promoting appreciation for nature.

## 1 Introduction

Despite politicians’, nongovernmental organizations’ (NGOs’), and activist groups’ many public calls about the urgent need to mitigate climate change, there is still a lack of engagement in environmental protection in nearly every society worldwide. This situation is analogous to an oversized classroom—designed for the dissemination of information—in which students appear to be limited in their capacity to absorb information from the learning opportunities to which they are exposed. Environmental attitude has been identified as a decisive personal reason behind learning and the remnants of learning, that is, the amount of knowledge students gain and retain (see Baierl et al., 2022a; Taube et al., 2021). In other words, and quite in contrast to traditional beliefs in education (e.g., Ballantyne and Packer, 1996; Fietkau and Kessel, 1981), environmental attitude is a main determinant of the reception and retention of information rather than the other way around, whereby information reinforces a preexisting environmental attitude. In light of this evidence on the effects of environmental attitude, it is important to identify means for increasing people’s environmental attitudes.

One line of research found that individuals who view nature as a backdrop for escaping from daily demands and for restorative experiences engage in more environmental protection (see Byrka et al., 2010; Hartig et al., 2001, 2007; Whitburn et al., 2020). A second line of research found evidence that attitude toward nature (i.e., people’s propensity to use and enjoy natural environments) and environmental attitude (i.e., people’s propensity to protect the environment) represent two distinct but positively correlated attitudes (see, e.g., Kaiser et al., 2013, 2014). Both lines of research are currently inconclusive regarding causality (Kaiser et al., 2014). Although typically not stated explicitly, researchers appear to agree that attitude toward nature determines environmental attitude (e.g., Dutcher et al., 2007). This view has received some support from research based on retrospective self-reflections (e.g., Chawla, 1997).

The objective of our research is to contribute to the ongoing investigation of the causal relationships involved in this phenomenon by extending Baierl et al.’s (2022a) model on the role of environmental attitude in facilitating learning about environmental issues. To this end, we employed the logic of mediation tests (see Baron and Kenny, 1986; Kenny et al., 1998) to ascertain whether environmental attitude mediates the influence of attitude toward nature on learning or whether the effect is in reverse.

### 1.1 Protecting versus enjoying natural environments

In this line of research, we view environmental attitude as a personal propensity to act in an environmentally protective way (Kaiser et al., 2010; Kaiser et al., 2007) or as people’s commitment to environmental protection. In this view, people’s attitudes represent a distinctly motivational concept (Allport, 1933). It is thereby unsurprising that there is a wealth of evidence, including behavioral observation, that environmental attitude is a significant predictor of manifest “behavior that harms the environment as little as possible” (Steg and Vlek, 2009, p. 309). For example, environmental attitude was found to be a significant predictor of sustainable travel behavior (e.g., Kaiser and Lange, 2021; Taube et al., 2018), energy consumption in a common’s dilemma (Kaiser and Byrka, 2015), green consumerism (Taube and Vetter, 2019), and vegetarian dish choices (Kaiser et al., 2020).

By contrast, attitude toward nature is viewed as a propensity that manifests in people’s appreciation for the natural environment (see Milfont and Duckitt, 2004). Accordingly, attitude toward nature can be derived from how people enjoy nature and use natural environments, such as visiting beautiful landscapes, engaging in outdoor sports, and other contemplative or restorative activities (e.g., Brügger et al., 2011; Tam, 2013). The particular attitude is more narrowly specified as an anthropocentric environmental attitude (and contrasted with an ecocentric attitude; see Thompson and Barton, 1994). More recently, the concept we refer to as attitude toward nature has been identified under various other names in the literature: for example, emotional affiliation with nature (Mayer and Frantz, 2004), personal connection to nature (Hinds and Sparks, 2008), relatedness with nature (Zelenski and Nisbet, 2014), environmental identity (Clayton, 2003), affinity toward nature (Kals et al., 1999), or people’s inclusion of nature in their self-concept (Schultz, 2002). Irrespective of the different names and conceptions, Brügger et al. (2011; see also Tam, 2013) corroborated a close convergence of measures of several of these concepts and their relevance for environmental protection. In this line of research, engagement in protecting the environment has typically been captured via self-reports (see, e.g., Clayton, 2003; Kals et al., 1999; Markowitz et al., 2012; Nisbet et al., 2008) but also via behavioral observations (see Whitburn et al., 2019) or observations of the consequences of behavior (i.e., energy consumption; see Frantz and Mayer, 2014).

In their research, Kaiser et al. (2013) were additionally able to distinguish attitude toward nature (i.e., the propensity to enjoy and use nature) from environmental attitude (i.e., the propensity to protect the environment). Simultaneously, they identified a strong positive relationship between the two attitudes of about *r* = 0.50 when corrected for measurement error attenuation (see also Kaiser et al., 2014). Such positive correlations have been reported reliably, although not always to this magnitude (see, e.g., Baldi et al., 2021; Davis et al., 2009; DeVille et al., 2021; Dutcher et al., 2007; Geng et al., 2015; Mackay and Schmitt, 2019).

Theoretically, it is typically expected that the personal benefits that come from using nature for restorative, recreational, spiritual, and other purposes create a dependency on natural environments to maintain the corresponding activities. Such a dependency expectedly renders people more concerned about and likely to put effort into environmental protection. In other words, the use and enjoyment of nature may, in fact, represent genuine reasons for people’s propensity to protect the environment. Such a view logically involves mediation: that is, the relevance of attitude toward nature for protecting the environment is transmitted via environmental attitude (Whitburn et al., 2020). Thus, strengthening people’s attitude toward nature by having them engage with and experience nature might present an avenue for additionally fostering their environmental attitude (e.g., Chawla, 1997; Dutcher et al., 2007; Ewert et al., 2005).

This line of primarily correlational research has yielded inconclusive results on the directionality of the relationship between these two attitudes and in a lack of evidence that substantiates a causal link between them (e.g., Kaiser et al., 2014; Whitburn et al., 2019). In addition, some studies have prompted skepticism about the mediating effects of environmental attitude, as attitude toward nature has been found to have a stronger effect on behavior than environmental attitude (see, e.g., Davis et al., 2009; Markowitz et al., 2012; Nisbet et al., 2008; Schultz, 2001: Study 3; Stets and Biga, 2003).

### 1.2 The two attitudes’ roles in learning about environmental issues

Schools serve a vital function in environmental education, as they educate and reach large numbers of students, particularly during formative periods of development (e.g., Otto et al., 2019). Schools provide opportunities for students to learn about the intricacies of environmental problems and to develop the competencies to combat these problems. Expectedly, people will consistently engage in environmental protection and, for example, avoid plastics only if they know about the detrimental consequences of plastic pollution and how to avoid plastics in their daily lives. Thus, it is crucial for students to acquire facts and develop competencies in order to learn how to protect the environment.

**Figure 1:**
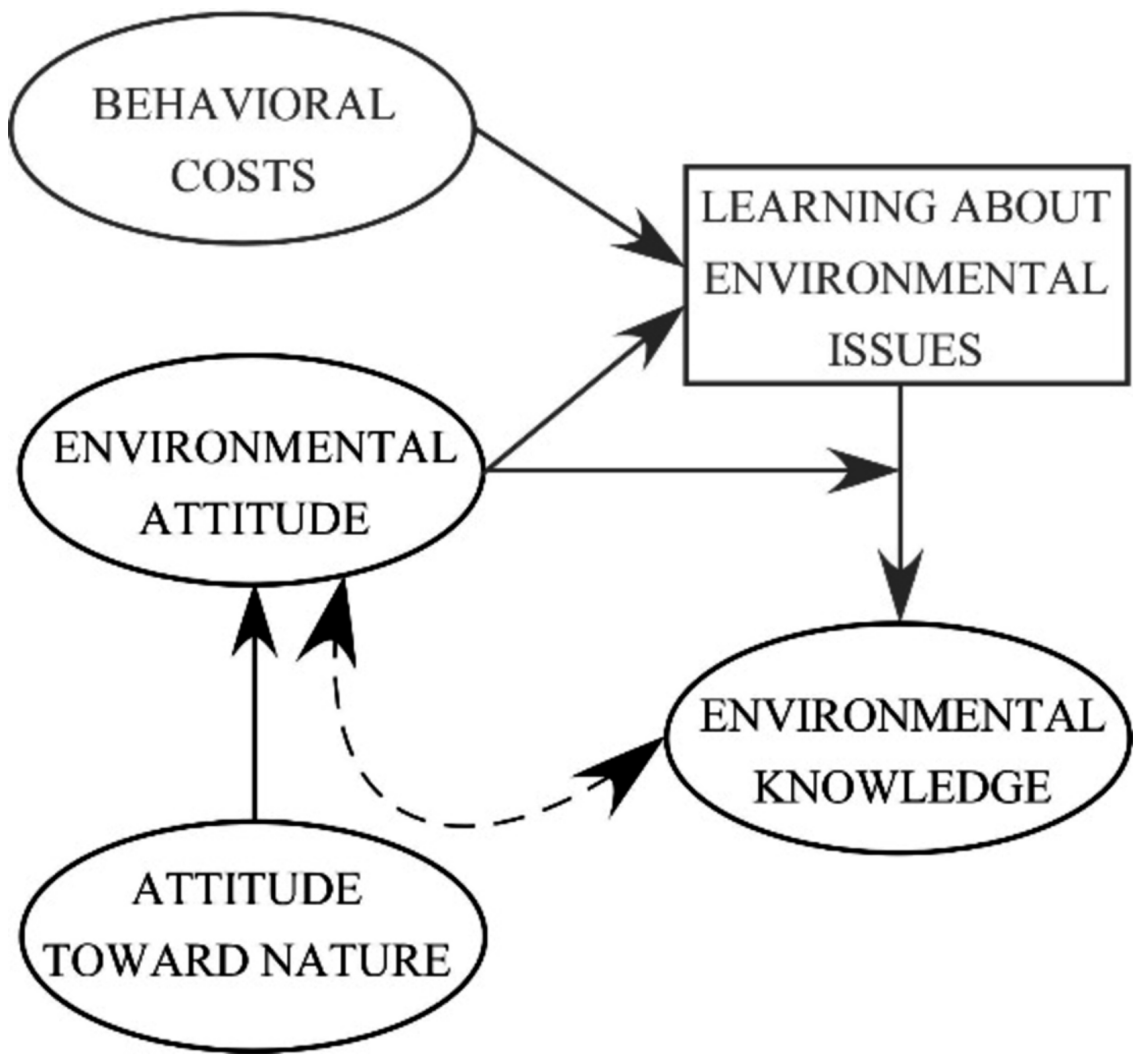
The hypothesized roles that environmental attitude and attitude toward nature play in learning; adapted from Baierl et al. (2022a). Arrows indicate directed relationships, whereas the double-headed arrow indicates the positive correlation between the consequence of learning (i.e., knowledge) and people’s attitudes. The rectangle represents behavioral manifestations of learning about environmental issues. The ovals represent latent variables.

Previous research has demonstrated that environmental attitude’s supportive role in learning about environmental issues manifests in two distinct ways (Baierl et al., 2022a): (a) People’s environmental attitudes and the costs of a specific learning activity (e.g., reading a text with new information) jointly affect learning (e.g., the likelihood that a text will be read); and (b) environmental attitude additionally affects the rigor with which people learn. By doing so, environmental attitude affects the intensity with which students seize their learning opportunities, subsequently determining the amount of knowledge they gain and later retain. Thus, we propose that individuals with stronger environmental attitudes not only seize more learning opportunities but also engage more rigorously in learning (see Figure 1). Consequently, the varied levels of environmental attitude determine the diverse learning outcomes typically found in classrooms.

In line with Dutcher et al. (2007), we expect that developing an appreciation for nature and, consequently, a propensity to use and enjoy nature fuels the propensity to protect the environment (and not the reverse). In other words, environmental attitude mediates the effects of attitude toward nature on behavior and on the consequences of behavior (e.g., people’s CO_2_ emissions or the amount of energy they consume). We argue that knowledge is the behavioral consequence that is easiest to measure. The more rigorously students seize learning opportunities, the more knowledge about environmental issues students gain and retain (see Figure 1).

### 1.3 Research goals

By extending Baierl et al.’s (2022a) model on the mediating role of environmental attitude in the effect of attitude toward nature on learning about environmental issues (see Figure 1), we address an unanswered question about causality. The objective of this research is to make a modest contribution to addressing this question by testing whether environmental attitude mediates the effect of attitude toward nature on the consequences of learning: that is, the knowledge that people eventually acquire.

**Figure 2:**
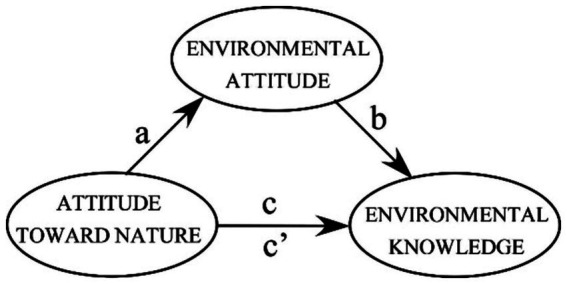
The schematic depiction of a mediation test in which environmental attitude mediates the effect of attitude toward nature on the consequences of learning about environmental issues (i.e., environmental knowledge).

On the basis of the logic of a conventional mediation test (see Baron and Kenny, 1986; Kenny et al., 1998), we first explore whether those who are more committed to using nature—people with stronger attitudes toward nature—also know more about environmental issues (Path c in Figure 2). With a view to the mediating role of environmental attitude, we expect that attitude toward nature affects environmental attitude (Path a in Figure 2) and that environmental attitude in return affects knowledge about environmental issues (Path b in Figure 2). Once the two mediating Paths a and b are added to the analysis, the strength of the effect of attitude toward nature on knowledge about environmental issues becomes nonsignificant (or at least significantly reduced: Path c’ is substantially smaller than Path c in Figure 2).

As the prototypical attitude toward nature measure was initially developed for adults (see, e.g., Brügger et al., 2011; Kaiser et al., 2013), we additionally explored whether the indicators that have been used so far would also be suitable for adolescents and young adults—a target group that has been relatively neglected in the study of attitude toward nature and its measurement (e.g., Barrera-Hernández et al., 2020; Cheng and Monroe, 2012).

## 2 Materials and methods

### 2.1 Participants and procedure

We used data from 1,486 students (*M*_age_ = 15.25, *SD* = 3.2) to address our research questions. Most were middle and high school students [*n* = 1,309; *M*_age_ = 14.44, *SD* = 1.82; 646 (49.4%) female and 592 (45.2%) male adolescents; 51 provided no information on gender], whereas 166 were university students [*M*_age_ = 22.29, *SD* = 3.23; 72 (43.4%) women and 89 (53.6%) men; five provided no information on gender]. For the total cohort, gender was almost evenly distributed, with 48.5% (*n* = 721) female and 46.2% (*n* = 686) male students.

The two attitude measures (i.e., environmental attitude and attitude toward nature) were administered to a total of 1,104 students [*M*_age_ = 14.43, *SD* = 2.19; 555 female (50.3%), 485 male (44.8%), and 42 who did not disclose a binary gender (3.8%)]. The remaining 382 students (*M*_age_ = 17.87, *SD* = 3.20; 43.5% female, 52.6% male, and 3.9% who did not disclose a binary gender) additionally participated in an educational intervention about the forest ecosystem and related sustainability issues. These students additionally received a test of environmental knowledge before and 6 weeks after the intervention to determine their gains in knowledge.

The 220-min educational intervention was designed as a student-centered, collaborative escape game in a typical classroom setting. With the goal of saving the forest, the students had to master the tasks embedded in nine workstations. The tasks were designed to help students learn facts about the forest ecosystem (e.g., flora and fauna biodiversity, succession of plant communities, and ecosystem services), sustainable behaviors to protect and conserve forests (e.g., recycling and considering eco-labels), and the environmental effects of land use and forest management strategies (e.g., deforestation and tree cultivation). For additional information regarding the intervention, see Fleissner-Martin and Bogner (2024).

The data were collected in schools and universities in Bavaria, Germany, in 2022 during regular lessons. The Bavarian Ministry of Education granted ethics approval for the survey. All principals of the participating schools were duly informed, and legal guardians provided written consent for all students below the age of 18 to take part in the study.

### 2.2 Measures

We measured environmental attitude, attitude toward nature, and environmental knowledge. Whereas the two attitude measures were administered to all participants (*N* = 1,486), the knowledge test was administered exclusively to participants in the educational intervention (*n* = 382). No items were excluded for any of the specific scale calibrations.

*Environmental attitude* reflects people’s propensity to protect the environment. The specific measure we employed is a variant, designed for adolescents (see Kaiser et al., 2007), of a well-established instrument for adults (see Kaiser and Wilson, 2004). This variant consists of 55 items: 15 opinions about environmental preservation, such as “We must set aside areas to protect endangered species,” and 40 self-reports of environmentally protective behaviors, such as “I collect and recycle used paper” (for more details, see Baierl et al., 2022a).

Participants answered the 55 polytomous items using either a 5-point frequency format ranging from 1 (*never*) to 5 (*always*) or a 5-point Likert scale ranging from 1 (*strongly disagree*) to 5 (*strongly agree*). Of the 55 items, 22 were negatively formulated. All these negatively formulated items, which represent a lack of the propensity to protect the environment, were reverse-coded before we calibrated the Rasch scale.

The measurement instrument was calibrated using a dichotomous Rasch model (for more details, see Rasch, 1960; for recent accounts, see, e.g., Bond et al., 2021; Wilson, 2023). Therefore, the items had to be converted into a dichotomous format. Prior to calibration, it is recommended that the absolute number of response options be reduced to prevent unreliable measurement due to excessive measurement error (for details and supporting evidence, see Kaiser and Lange, 2021). This dichotomization is distinct from the untenable dichotomization of person scores after establishing an attitude scale as a continuous measure (DeCoster et al., 2009). For the dichotomization, the responses *never*, *seldom*, *occasionally*, *strongly disagree*, *disagree*, and *not sure*/*neutral* were coded as expressions of a lack of the propensity to protect the environment (0). The responses *often*, *always*, *agree*, and *strongly agree* were coded as expressions of the propensity to protect the environment (1).

Due to limitations in in-class time, approximately half of the sample (*n* = 718) were presented with only 25 of the 55 items that would have been available for measuring environmental attitude in principle. Therefore, 718 participants responded to fewer items by design. Such a reduction in items can negatively affect an instrument’s reliability by generally inflating the standard error of measurement. Nevertheless, the separation reliability indicated that the scale was effective in differentiating between students (*rel* = 0.73).

Similarly, the fit values reflecting the discrepancy between the Rasch model’s predicted and the observed responses were also encouraging. Specifically, mean square (*MS_w_*) values weighted by the item variance fell between 0.84 and 1.21 (for more details, see, e.g., Wright and Masters, 1982). An *MS_w_* value of 1.21 indicates 21% more response variation than the model predicted. It was reasonable to conclude that the Rasch-model-based expectations provided an adequate fit to the actual responses (for reference values and their justification, see Wright et al., 1994) and that the calibration of the scale was in line with previous such calibrations (e.g., Baierl et al., 2022a; Kaiser et al., 2007).

Environmental attitude measurements ranged from −4.07 to 4.40 logits (*M* = −0.50, *SD* = 0.91). Logits represent the natural logarithm of the ratio between environmentally protective and nonprotective answers such that a more positive logit value indicates a stronger attitude.

*Attitude toward nature* was measured with 21 self-reports of uses of nature for restorative and recreational experiences, such as “I take time to watch the clouds pass by,” and with 20 opinions expressing appreciation for nature, such as “I like the quiet of nature” (for the complete list of items, see Supplementary Table S4). Whereas evaluative statements are typical indicators of attitudes, they are often susceptible to some people’s socially desirable responses and to excessively high approval ratings (i.e., ceiling effects). By contrast, self-reported behaviors focus on past performance and are typically more difficult to endorse. People’s attitudes can also be inferred from records of past behavior because individuals often engage in behavior with intentions (to achieve specific goals). For example, a person might watch stars while going outside to smoke or, alternatively, to contemplate the night sky. By collecting behavioral self-reports that capture the extents to which people endorse particular goals (e.g., using and enjoying nature), it is thereby also possible to derive their corresponding attitudes.

Most of the items originated from Brügger et al. (2011) and, thus, from the adult version of the proposed measure. Four evaluative statements originated from Bogner and Wiseman (2006). Of the 41 items, 8 were adapted for broader use. For example, “As a child, I spent time in the woods” was reformulated as “As a child, I spent time outdoors.”

As with the environmental attitude items, participants answered the 20 polytomous items using either a 5-point frequency scale (*i* = 11) ranging from 1 (*never*) to 5 (*always*) or a 5-point Likert scale (*j* = 9) ranging from 1 (*strongly disagree*) to 5 (*strongly agree*). Each of the 21 dichotomous items was answered in a yes-no format. Participants could answer with *not applicable* if they could not provide an answer. Those answers were coded as missing values. Three items were negatively formulated and reverse-coded before the analysis. Once again, polytomous items were converted into a dichotomous format to prevent excessive measurement error. For the dichotomization, the responses *never*, *seldom*, *occasionally*, *strongly disagree*, *disagree*, and *not sure*/*neutral* were coded as expressions of a lack of the propensity to use and enjoy nature (0). The responses *often*, *always*, *agree*, and *strongly agree* were coded as expressions of the propensity to use and enjoy nature (1). Details about the calibration of the measurement instrument with the dichotomous Rasch model are presented in the Results section.

*Environmental knowledge* was measured with 25 knowledge items. These items were presented in a multiple-choice format with one correct answer and three distractors. The knowledge items covered facts about ecosystems and environmental issues, proper behavior that does not harm the ecosystem, and the ecological impact of specific technologies or landscape/forest management strategies. An example of facts is the following: “Abiotic factors are” (a) “water and light,” (b) “predators and competitors,” (c) “human beings and the industry,” and (d) “minerals and sustenance.” An example of behavior options is the following: “Identify the paper product that can be recycled by placing it in the paper trash.” The four options are: (a) “handkerchief,” (b) “egg crate,” (c) “public transport ticket,” and (d) “baking paper.” An example of an item that addresses ecological impacts is: “Identify the option that is *not* a consequence of deforestation.” The four options are: (a) “avalanche risk increases,” (b) “earthquake risk increases,” (c) “flooding increases,” and (d) “rockfall risk increases.” For more details about the knowledge items, see Fleissner-Martin et al. (2023).

We calibrated the environmental knowledge measure as a dichotomous Rasch scale. Environmental knowledge ranged from −2.35 and 3.61 logits (*M* = 0.53, *SD* = 0.89). This time, logits represented the natural logarithm of the ratio between correct and incorrect answers with more positive logit values indicating more knowledge. Additionally, the separation reliability indicated that the scale was able to differentiate students on the basis of their knowledge levels (*rel* = 0.71). Furthermore, mean square (*MS_w_*) values weighted by the item variance between 0.85 and 1.25 confirmed that the scale was satisfactory and in alignment with previous impromptu measures of knowledge (see, e.g., Baierl et al., 2022b).

## 3 Results

We report the results in two sections: In the first section, we report the psychometric features of the proposed Attitude Toward Nature scale. In the second section, we report results on our mediation test in which environmental attitude mediates the effect of attitude toward nature on learning about environmental issues (see Figure 2).

### 3.1 Measurement of adolescents’ attitudes toward nature

The 41 items were calibrated with the dichotomous Rasch model. The separation reliability indicated that the scale differentiated between people (*rel* = 0.87). Similarly acceptable were the fit values that reflected the discrepancy between the Rasch model’s predicted responses and the observed responses. Because our sample of 1,486 respondents was relatively large, we assessed item fit with Mean Square (*MS_w_*) values weighted by the item variance. An *MS_w_* value of 0.75 corresponds to 25% less than the expected amount of variation, and an *MS_w_* value of 1.33 indicates 33% more variation in the data than what was predicted by the measurement model. *MS_w_* values in this approximate range are considered a sensible threshold for instruments used in the scientific exploration of empirical relationships (see Wright et al., 1994). The item fit values for the current study ranged from 0.73 to 1.20 *MS_w_*. For a complete list of items, their difficulties, and their *MS_w_* values, see Supplementary Table S4.

Attitude toward nature values fell between −4.17 and 3.44 logits (*M* = −0.65, *SD* = 1.17), and items ranged from −1.69 to 2.27 logits. Logits represent the typical units of Rasch scales. Logits were used to measure both people’s attitudes and the item difficulties. Logits represent the natural logarithm of the ratio between affirmative and negative answers. More positive logit values correspond with the strength of people’s attitudes and the difficulty of an affirmative response to an item.

**Figure 3:**
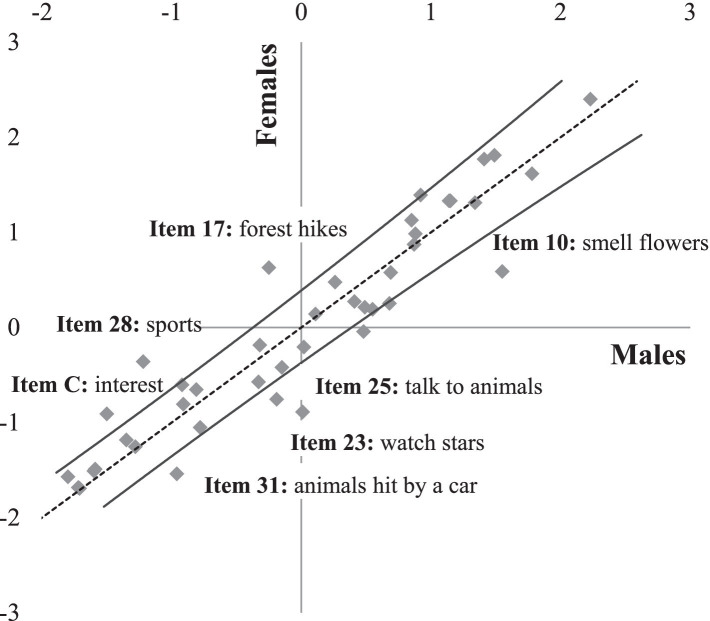
Comparison of item difficulties between female (*n* = 721) and male students (*n* = 686). Each square represents one of 41 items used in the measurement of attitude toward nature. The dashed line represents the identity line where all behaviors would lie if the item difficulties were perfectly comparable for the two genders. The 95% confidence interval (solid black lines) statistically factors in uncertainty when comparing item difficulties (for more details, see Bond et al., 2021; Wright and Stone, 1999). Item numbers refer to the Supplementary Table S4.

As measurements of people should be unbiased (i.e., fair; see, e.g., Fischer, 1987), instruments that are applied to make numerical comparisons between people should not unduly benefit members of certain groups. For example, bias occurs when men are more likely than women to provide affirmative responses to the items on a measurement instrument. To appreciate what is sometimes called specific objectivity in the proposed measurement instrument, we contrasted [with a test for differential item functioning (DIF)] the likelihoods of affirmative responses to the 41 items used in the measurement of attitude toward nature for the two gender groups (see Figure 3). The item difficulties were derived from calibrating two specific scales, one for female students (*rel* = 0.87, *n* = 721) and one for male students (*rel* = 0.85, *n* = 686). For male and female students, most of the item fit values fell within an acceptable range from 0.71 to 1.28 *MS_w_*.

Of the 41 items, seven (17.1%) fell conspicuously outside the 95% confidence interval (the solid black lines) that we used to statistically factor uncertainty into the comparison of item difficulties (see Figure 3). Affirmative responses to items were either more difficult for females (above the confidence interval) or more difficult for males (below the confidence interval). Of the seven biased items, similar numbers benefitted female (*i* = 4) and male students (*j* = 3). In other words, biased items that fell above or below the confidence interval were relatively evenly arranged. Figure 3 illustrates this pattern and, thus, evidences a relatively gender-fair measurement of attitude toward nature. This conclusion was empirically backed by a Pearson correlation between the item difficulties for males and females of *r* = 0.94 (*p* < 0.001). Numerical details can be found in Supplementary Table S1.

To test our conclusion about gender fairness, we calibrated two different Attitude Toward Nature scales: one for female and one for male students. This time, the seven biased items were estimated separately for male and female students, whereas the difficulties of the other 34 items were fixed to equality for the two gender groups. Results showed no differences in the numerical depictions of people’s attitudes toward nature with Pearson correlations of *r* = 1.0 (*n* = 721) and *r* = 1.0 (*n* = 686) between the two measures: one ignoring and one considering DIF between male and female students.

In addition to gender, individuals in different phases of their adolescence might also benefit from differential difficulties in affirmative responses to items, for example, when individuals implement their personal propensities to use and enjoy nature restoratively or recreationally in different ways at different ages. Accordingly, we ran separate Rasch model tests for 11-13-year-olds (*rel* = 0.82, *n* = 349), 14-15-year-olds (*rel* = 0.86, *n* = 622), and 16-18-year-olds (*rel* = 0.88, *n* = 338). For 14-15-year-olds, the item fit values were in an acceptable range between 0.75 and 1.19 *MS_w_*. By contrast, we found five items for 11-13-year-olds and two items for 16-18-year-olds that fell outside the acceptable range for fit (see Table 1).

**Table 1:** Potentially problematic items for 11-13-year-olds (Items #1–#5) and for 16-18-year-olds (Items #6–#7).

		δ (SEM)	*MS*_w_	*MS*_u_
19	I spend time in a park.	−1.11 (0.12)	1.33	1.52
4	*I would always prefer spending time with my friends to being alone in nature.*	0.95 (0.18)	1.32	1.96
7	I watch TV shows that have animals as the main characters.	−0.45 (0.13)	1.29	1.28
A	*I like a grass lawn more than a place where flowers grow independently.*	−0.19 (0.14)	1.26	1.37
26	Even when it is very cold or rainy, I go out for a walk.	−2.14 (0.13)	1.21	1.48
36	*The noise of animals gets on my nerves*.	−1.76 (0.13)	1.24	1.38
A	*I like a grass lawn more than a place where flowers grow independently.*	0.93 (0.15)	1.21	2.31

Items in italics were negatively formulated and reverse-coded before the statistical analyses. Item difficulties (δ) and standard errors of measurement (SEM) are expressed in logits. Mean square (MS) values—unweighted (u) and weighted (_w_) by the item variance—reflect the relative discrepancy between the model’s predicted responses and the actual responses used to assess item fit. Item numbers refer to the Supplementary Table S4.

When we contrasted 11-13-year-olds, 14-15-year-olds, and 16-18-year-olds in two DIF tests, we additionally found considerable DIF (see Supplementary Tables S2, S3). Of the 41 items, 25 were biased to the advantage of either 11-13-year-olds (*i* = 10) or 14-15-year-olds (*j* = 15). That is, 14-15-year-olds benefited slightly more from the selected items, as more items seemed better suited to reflect how 14-15-year-olds express their propensity to use and enjoy nature (for more details, see Supplementary Table S2). As age increases, verbally expressing appreciation for nature (but less so for actual outdoor activities) appears to be the preferred way to express attitudes toward nature.

Of the 41 items, 12 were biased to the advantage of 14-15-year-olds (*i* = 5) or 16-18-year-olds (*j* = 7). Again, the 12 biased items were relatively evenly arranged, with similar numbers benefiting 16-18-year-olds (*i* = 7) and 14-15-year-olds (*j* = 5: for more details, Supplementary Table S3). At older ages, contemplative outdoor activities appeared to have become the relatively preferred way to express one’s propensity to use and enjoy nature.

To test our conclusion regarding age fairness, we calibrated three different Attitude Toward Nature scales: one for 14-15-year-olds (*rel* = 0.82), one for 14-15-year-olds (*rel* = 0.86), and one for 16-18-year-olds (*rel* = 0.88). In these scale calibrations, the 12 items with the most extreme bias were estimated separately in the different age groups, whereas the difficulties of the remaining 29 items were fixed to equality for the three age groups. This time, item fit values across the three age groups were all in an acceptable range, with *MS_w_* values between 0.72 and 1.21. Results showed marginal differences in the numerical depictions of students’ attitude toward nature with Pearson correlations of *r* = 1.0 (*n* = 349), *r* = 0.99 (*n* = 622), and *r* = 0.99 (*n* = 338) between the two measures: one ignoring and one considering DIF across the three age groups.

### 3.2 Environmental attitude as the mediator of attitude toward nature

**Figure 4:**
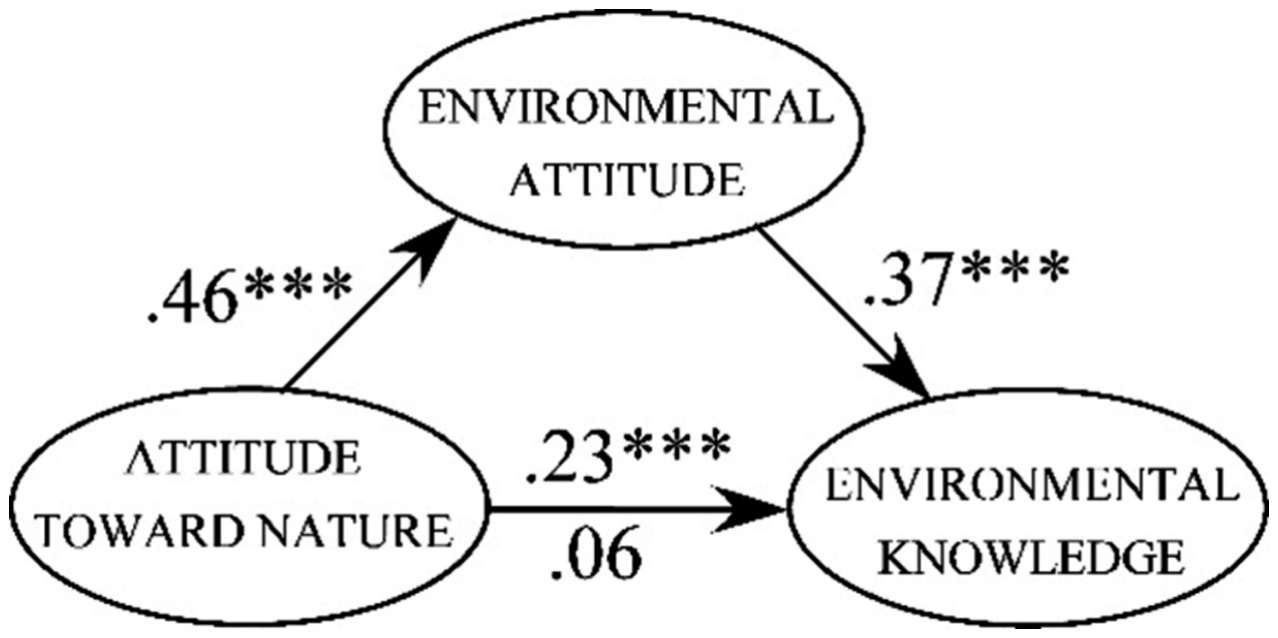
The effect of attitude toward nature on the consequences of learning: the environmental knowledge levels students retained at follow-up 6 weeks after the intervention. The effect was no longer significant when environmental attitude was included as the mediator. ***p < 0.001.

In our analysis of the 382 students who participated in the educational intervention, we followed the procedure for mediation tests proposed by Baron and Kenny (1986) (see Figure 2). Because of its superior technical features, we employed Preacher and Hayes’ bootstrapping method (see, e.g., MacKinnon et al., 2002; Preacher et al., 2007). Initially, we established that those with stronger attitudes toward nature also knew more about environmental issues (Path c in Figure 2) after the intervention (b = 0.23, 95% CI [0.13, 0.33], *β* = 0.23). Once we added the mediator (i.e., environmental attitude) by including the mediating Paths a (b = 0.42, 95% CI [0.34, 0.50], *β* = 0.46) and b (b = 0.41, 95% CI [0.29, 0.52], *β* = 0.37) in the analysis, the strength of the effect of attitude toward nature on knowledge about environmental issues (Path c’) was no longer significant (b = 0.06, 95% CI [−0.04, 0.18], *β* = 0.06: see Figure 4).

To gain a more profound understanding of the role of attitude toward nature in learning and the hypothesized relationships depicted in Figure 1, we initially verified that the intervention was beneficial for students. Apparent in the mean values, knowledge levels increased significantly from the assessment conducted before the intervention (*M_pre_* = 0.51, *SD* = 0.77) to the assessment conducted 6 weeks after the intervention (*M_retention_* = 0.76, *SD* = 1.03: *p* < 0.001; *n* = 382). In addition to students’ mean knowledge levels, the standard deviation of students’ knowledge also increased, thus indicating a disparity in learning. In other words, not all students benefited equally; some students learned disproportionally more than others. Indeed, students with increasing levels of attitude toward nature gradually gained more knowledge (*β* = 0.23): *F*_(1, 381)_ = 21.90, *p* < 0.001, *R*^2^ = 0.05.

The relatively superior learning was also reflected by the correlations between students’ attitude toward nature and their preexisting (*r* = 0.19, *p* < 0.001, *n* = 382) and retained knowledge (*r* = 0.24, *p* < 0.001, *n* = 382), which—although not in an inference statistical sense—increased in strength in a descriptive sense after the intervention. Finally, we additionally corroborated that knowledge gains (i.e., the difference between retained and prior knowledge) were, in fact, controlled by students’ attitude toward nature (*β* = 0.10): *F*_(1, 381)_ = 3.92, *p* = 0.048, *R*^2^ = 0.01.

Against the backdrop of the hypothesized mediating role of environmental attitude (see Figure 4), we revisited the attitude effects and replaced attitude toward nature with environmental attitude. At least on a descriptive level, we found that the measures of effect size (i.e., *r* and *R*^2^) became quantitatively larger than the equivalent effects with attitude toward nature. Evidentially, students with increasing levels of environmental attitude also gained linearly more knowledge (*β* = 0.41): *F*_(1, 381)_ = 76.31, *p* < 0.001, *R*^2^ = 0.17. Whereas the connection between environmental attitude and knowledge remained relatively comparable for both retained knowledge (*r* = 0.39, *p* < 0.001, *n* = 382) and preexisting knowledge (*r* = 0.40, *p* < 0.001, *n* = 382), they almost doubled in magnitude. And once more, the relatively superior learning effect was again reflected in the knowledge gains (i.e., the difference between retained and prior knowledge) that were determined by students’ attitude, this time environmental attitude (*β* = 0.15): *F*_(1, 381)_ = 8.31, *p* = 0.004, *R*^2^ = 0.02.

## 4 Discussion

We found that attitude toward nature and environmental attitude were both relevant for learning about environmental issues, which in turn is likely also critical for further engagement in environmental protection (see also, e.g., Mackay and Schmitt, 2019). People who are inclined to use and enjoy nature or to protect the environment learn more and, thus, subsequently know comparatively more about environmental issues. We suspect that similar effects can be found with out-of-classroom types of information dissemination, such as public campaigns. The effect of attitude toward nature on knowledge was, as hypothesized, fully mediated by environmental attitude. With our findings, we provide a first tentative, previously missing piece of circumstantial evidence that supports the causal process depicted in Figure 1. We replicated the relationship between the two attitude measures of around *r_corr_* = 0.50 typically found in adults (when corrected for measurement error attenuation; see, e.g., Kaiser et al., 2013, 2014; Mayer and Frantz, 2004; Nisbet et al., 2008) in a sample of adolescents (*r_corr_* = 0.49, *N* = 1,399).

Our mediation test yielded some novel but still circumstantial evidence for the presumed causal link between attitude toward nature and environmental attitude (see Figure 1). The opposite view of environmental attitude as the cause of nature appreciation and engagement (see Soga and Gaston, 2016) has thereby lost some credibility. While our findings are promising, more robust evidence for causation is needed, particularly with larger samples and more rigorous criteria, for example, via dose–response gradients (see, e.g., Hill, 1965) or by furthering students’ attitudes toward nature through education. To achieve such ends, valid measures of both environmental attitude and attitude toward nature are needed.

Although the students in this study gained substantial knowledge about the environment and environmental protection, we did not evaluate whether the in-class education intervention improved their attitude scores. In regard to developing an instrument for measuring attitude toward nature in adolescents, our findings indicate that developing a sound measure of attitude toward nature for adolescents is a more challenging endeavor than suggested by the currently available one-for-all instruments used with adults (see, e.g., Clayton, 2003; Hinds and Sparks, 2008; Brügger et al., 2011).

In line with Brügger et al. (2011), we employed the Campbell paradigm (see Kaiser et al., 2010) to measure attitude toward nature. Additionally, we adopted 37 of 40 of Brügger et al.’s (2011) items originally designed for adults in our sample of adolescents. Unexpectedly, we discovered that adolescents at different ages implement their personal attitude toward nature in different ways. This tendency could be seen in some substantive DIF, which indicates that different activities and verbal expressions of opinions appeal to adolescents of different ages to different degrees.

In contrast to 11-13-year-olds, 14-15-year-olds did not reveal their attitude toward nature by engaging in outdoor activities. Instead, they were more likely to express favorable opinions about nature and its use, for example, by stressing that animals are interesting or that pets are part of the family. For 16-18-year-olds (in contrast to 14-15-year-olds), contemplative outdoor activities—watching dragonflies or stars at night—represented their preferred way to reveal their attitude toward nature (for more details, see Supplementary Tables S2, S3).

In summary, younger adolescents preferred exploratory, active, and immersive engagement in and with nature (see also Bonnett and Williams, 1998). By contrast, older adolescents were more contemplative and nature-receptive and indicated stronger emotional responses to nature. Although adolescence is well-known as a transitive period of change, our research provides initial evidence that this transitive period can also be captured by how adolescents reveal their personal attitude toward nature across different age groups. Future research is needed to establish a more solid understanding of these differences.

In contrast to the age-specific ways in which adolescents express their attitudes toward nature, gender differences in how adolescents express their attitudes toward nature were much less pronounced (see Figure 3). For instance, male adolescents were more likely to express favorable opinions about using nature, for example, by expressing that they prefer forest hikes to city strolls or outdoor to indoor sports. For female adolescents, by contrast, some of the contemplative activities seemed comparatively more attractive, for example, smelling flowers, talking to animals, and gazing at stars. In light of the aforementioned evidence, it can be concluded that the gender-specific differences did not severely impede the validity of the proposed measurement instrument in assessing adolescents’ attitudes toward nature.

By contrast, the age-specific differences are serious enough to impede the validity of the proposed measurement instrument when there is a need to compare adolescents’ attitudes toward nature across different age groups. These differences were reflected in the relatively poor fit statistics of seven items that were not effective indicators of adolescents’ attitudes toward nature in two of the three age groups (see Table 1). When the age-group bias of items was considered in the scale calibration—with a combination of age-group-specific and uniform item difficulties—the fit statistics of the problematic items from Table 1 improved relative to a scale calibration when all item difficulties were uniform for all age groups.

The differential functioning of items for different age groups needs to be accounted for; otherwise, the suggested measurement approach cannot validly reflect every adolescent’s attitude toward nature. The DIF can be accounted for by applying age-group-specific calibrations and, preferably, by employing some age-group-specific attitude-toward-nature measures that build on the items demonstrating good functioning for all age groups (Supplementary Table S4). This core item set represents the backbone of the valid and comprehensive future measurement of attitude toward nature.

With our work, we extended knowledge about the role of environmental attitude in learning and in the actual learning process in environmental protection research. People’s propensity to use and enjoy nature likely leads to a greater propensity to protect the environment (and not the other way around). Therefore, both attitudes—that is, attitude toward nature and environmental attitude—are, in principle, suitable targets for formal and informal education. Next to educational programs meant to foster environmental attitude (see, e.g., Hartley et al., 2015), attitude toward nature might be another more accessible target that makes it possible to take an experiential approach to helping students learn about sustainable development and other environmental issues: for example, via personal contact with nature (by caring for animals or plants), outdoor activities in nature, nature walks, or zoo visits (see, e.g., Baierl et al., 2022b; Dresner and Gill, 1994; Duerden and Witt, 2010; Van Matre and Johnson, 1988).

## Data Availability

The datasets presented in this article are not readily available because data cannot be shared publicly because of the Bavarian Ministry for Education’s guidelines regarding data of underaged participants. Requests to access the datasets should be directed to tessa-marie.baierl@uni-bayreuth.de.
