# Supplementary material for: The role of attitude toward nature in learning about environmental issues
Source: Front Psychol. 2024 Nov 13;15:1471026. doi: 10.3389/fpsyg.2024.1471026 (PMC11613741; doi:10.3389/fpsyg.2024.1471026)
Supplement: Supplementary file 1 [file Data_Sheet_1.pdf]

Supplementary Material

Table S1: Attitude toward nature items that differed significantly in their difficulties (δ) between female and male students.

	Item	δ_{males} (*SD*)	$\delta_{females}$ (*SD*)	*p*
1	I deliberately take time to watch the stars at night.	−0.01 (0.09)	**−0.87 (0.09)**	< .001
2	I prefer outdoor to indoor sports.	**−1.22 (0.09)**	−0.35 (0.09)	< .001
3	I prefer forest hikes to city strolls.	**−0.25 (0.10)**	0.62 (0.10)	< .001
4	I consciously take time to smell flowers.	1.55 (0.13)	**0.58 (0.09)**	< .001
5	It is interesting to know what kinds of creatures live in ponds or rivers.	**-1.50 (0.09)**	−0.89 (0.09)	< .001
6	I talk to animals.	-0.19 (0.09)	**−0.74 (0.09)**	< .001
7	It makes me miserable to see animals that were hit by a car.	−0.96 (0.09)	**−1.51 (0.09)**	< .001

Note: The numbers in **bold** indicate the gender group for which the item was less difficult and, consequently, received more affirmative responses.

Table S2: Attitude toward nature items that differed significantly in their difficulties (δ) between 11-13-year-olds and 14-15-year-olds. Items are listed according to DIF, from the largest to the smallest.

	Item	$\delta_{11\text{-}13\ years}$ (*SD*)	$\delta_{14\text{-}15\ years}$ (*SD*)	*p*
**1**	As a child, I spent time outdoors.	0.75 (0.17)	**−3.17 (0.17)**	< .001
**2**	Animals are interesting.	0.62 (0.16)	**−2.74 (0.15)**	< .001
**3**	I consciously watch or listen to birds.	**−0.81 (0.12)**	1.68 (0.12)	< .001
**4**	I feel the need to be out in nature.	**−2.14 (0.13)**	0.08 (0.09)	< .001
**5**	It makes me miserable to see animals that were hit by a car.	0.69 (0.17)	**−2.35 (0.13)**	< .001
**6**	I cross meadows barefoot.	**−1.35 (0.12)**	0.70 (0.10)	< .001
**7**	I mourn the loss of pets.	0.55 (0.16)	**−2.13 (0.12)**	< .001
8	I listen to and look at birds.	**-0.34 (0.13)**	2.03 (0.13)	< .001
9	Pets are part of the family.	0.63 (0.17)	**−1.91 (0.11)**	< .001
10	A cleared forest makes me miserable.	0.81 (0.17)	**−1.86 (0.12)**	< .001
11	I prefer outdoor to indoor sports.	0.76 (0.17)	**−1.86 (0.12)**	< .001
12	I talk to animals.	**−1.85 (0.12)**	−0.02 (0.09)	< .001
13	I watch TV shows that have animals as the main characters.	**−0.45 (0.13)**	1.62 (0.12)	< .001
**14**	I enjoy sitting at a pond watching dragonflies.	**−0.74 (0.12)**	1.10 (0.10)	< .001
15	I enjoy trips to the countryside.	**−2.90 (0.14)**	−1.11 (0.09)	< .001
**16**	*The noise of animals gets on my nerves.*	**−2.78 (0.14)**	−1.05 (0.09)	< .001
17	Listening to the sounds of nature makes me relax.	0.84 (0.17)	**−1.26 (0.12)**	< .001
18	I collect objects from nature such as stones or insects.	**−0.37 (0.13)**	1.27 (0.11)	< .001
19	I spend time in a park.	**−1.11 (0.12)**	0.32 (0.09)	< .001
20	It is interesting to know what kinds of creatures live in ponds or rivers.	**−2.11 (0.13)**	−0.68 (0.09)	< .001
21	Walking through nature makes me forget about my daily worries.	1.25 (0.20)	**−0.59 0.11**	< .001
**22**	I like the quiet of nature.	**−2.10**	-0.91	< .001

		**(0.13)**	(0.09)	
23	I personally take care of plants.	0.77 (0.17)	**−0.72 (0.11)**	< .001
24	I take time to watch clouds pass by.	**−0.50 (0.13)**	0.67 (0.10)	< .001
25	I help snails cross the street.	**0.22 (0.15)**	1.56 (0.12)	< .001

Note: The numbers in **bold** indicate the age group for which the item was less difficult and, consequently, received more affirmative responses. Items in italics were negatively formulated and reverse-coded before the statistical analyses.

Table S3: Attitude toward nature items that differed significantly in their difficulties (δ) between 14-15-year-olds and 16-18-year-olds. Items are listed according to DIF, from the largest to the smallest.

	Item	$\delta_{14\text{-}15\ years}$ (*SD*)	$\delta_{16\text{-}18\ years}$ (*SD*)	*p*
1	I feel the need to be out in nature.	0.08 (0.09)	**−0.95 (0.13)**	< .001
2	A cleared forest makes me miserable.	**−1.86 (0.12)**	−0.83 (0.13)	< .001
3	I like the quiet of nature.	−0.91 (0.09)	**−1.83 (0.14)**	< .001
4	I deliberately take time to watch the stars at night.	−0.06 (0.09)	**−0.83 (0.13)**	< .001
5	I prefer outdoor to indoor sports.	**−1.89 (0.13)**	−1.00 (0.13)	< .001
6	It makes me miserable to see animals that were hit by a car.	**−2.35 (0.13)**	−1.52 (0.14)	< .001
7	*The noise of animals gets on my nerves.*	−1.05 (0.09)	**−1.76 (0.13)**	< .001
8	Carving a tree feels like cutting myself.	**0.65 (0.12)**	1.60 (0.19)	< .001
9	I enjoy sitting at a pond watching dragonflies.	1.10 (0.10)	**0.39 (0.14)**	< .001
10	If an insect is in my home, such as a fly, I try to catch and release it rather than kill it.	**−0.28 (0.10)**	0.40 (0.14)	< .001
11	As a child, I spent time outdoors.	**−3.17 (0.17)**	−2.38 (0.15)	< .001
12	Animals are interesting.	**−2.74 (0.15)**	−2.01 (0.14)	< .001

Note: The numbers in **bold** indicate the age group for which the item was less difficult and, consequently, received more affirmative responses. Items in italics were negatively formulated and reverse-coded before the statistical analyses.

Table S4: Item difficulties (δ) and fit statistics for the items used in the measurement of attitude toward nature for the total cohort (all) and the three age groups

		δ (SEM)	MS_w all	MS_w 11-13y	MS_w 14-15y	MS_w 16-18y
1	I talk to plants.	2.27 (.10)	1.02	1.02	1.01	1.08
2	I get up early to watch the sunrise.	1.67 (.08)	1.08	1.14	1.02	1.00
3	I have audio recordings of the sounds of nature.	1.61 (.09)	1.02	0.83	1.07	1.00
4	*I would always prefer spending time with my friends to being alone in nature.*	1.58 (0.08)	1.20	1.32	1.19	1.16
5	I help snails cross the street.	1.30 (0.08)	1.05	1.03	0.96	1.05
6	I consciously watch or listen to birds.	1.23 (0.08)	1.04	1.00	0.98	0.99
7	I watch TV shows that have animals as the main characters.	1.19 (0.07)	1.20	1.29	1.00	1.10
8	Carving a tree feels like cutting myself.	1.18 (0.08)	0.91	0.79	0.95	0.87
9	I collect objects from nature, such as stones or insects.	0.96 (0.07)	1.16	1.18	1.02	1.05
10	I consciously take time to smell flowers.	0.92 (0.07)	0.98	1.01	1.00	0.92
11	I listen to and look at birds.	0.89 (0.07)	1.08	0.97	0.93	0.94
12	Indoor plants are part of the family.	0.88 (0.07)	0.97	0.88	1.04	1.01
A	*I like a grass lawn more than a place where flowers grow independently.*	0.59 (.06)	1.20	1.26	1.16	1.21
13	If one of my plants dies, I reproach myself.	0.45 (0.07)	0.84	0.78	0.93	0.88
14	I enjoy gardening.	0.40 (0.07)	0.80	0.71	0.75	0.84
15	My favorite place is in nature.	0.38 (0.07)	0.74	0.70	0.79	0.78
16	I take time to watch the clouds pass by.	0.33 (0.06)	1.03	1.0	0.98	0.99
B	I enjoy sitting by a pond watching dragonflies.	0.34 (0.06)	1.06	1.09	0.93	1.09
17	I prefer forest hikes to city strolls.	0.23 (0.07)	0.89	0.81	0.96	1.02
18	I cross meadows barefoot.	0.17 (0.06)	1.19	1.18	1.04	1.04
19	I spend time in a park.	-0.11 (0.06)	1.20	1.33	1.12	1.18
20	If an insect is in my home, such as a fly, I try to catch and release it rather than kill it.	0.13 (0.06)	0.87	0.83	0.92	0.96

		δ (SEM)	MS_w all	MS_w 11-13y	MS_w 14-15y	MS_w 16-18y
21	Listening to the sounds of animals makes me relax.	-0.16 (0.07)	0.73	0.70	0.75	0.84
22	Walking through nature makes me forget about my daily worries.	-0.24 (0.07)	0.79	0.79	0.88	0.84
23	I deliberately take time to watch the stars at night.	-0.47 (0.06)	1.09	1.10	1.05	1.05
24	I personally take care of plants.	-0.42 (0.06)	0.82	0.76	0.95	0.88
25	I talk to animals.	-0.50 (0.06)	1.19	1.08	1.11	1.12
26	Even when it is very cold or rainy, I go out for a walk.	-0.75 (0.06)	1.14	1.17	1.14	1.17
27	I feel the need to be out in nature.	-0.77 (0.06)	1.14	1.21	0.96	1.10
28	I prefer outdoor to indoor sports.	-0.78 (0.06)	1.00	0.90	1.08	1.09
29	Listening to the sounds of nature makes me relax.	-0.80 (0.06)	0.73	0.77	0.80	0.75
30	A cleared forest makes me miserable.	-0.86 (0.06)	0.76	0.72	0.82	0.79
31	It makes me miserable to see animals that were hit by a car.	-1.18 (0.06)	0.78	0.72	0.85	0.81
C	It is interesting to know what kinds of creatures live in ponds or rivers.	-1.20 (0.06)	1.16	1.10	1.12	1.19
32	I mourn the loss of pets.	-1.22 (0.06)	0.85	0.72	1.00	0.93
33	Pets are part of the family.	-1.22 (0.06)	0.90	0.79	1.10	0.98
34	Animals are exciting.	-1.49 (0.06)	0.77	0.73	0.86	0.79
D	I like the quiet of nature.	-1.52 (0.06)	1.06	1.09	0.99	1.09
35	As a child, I spent time outdoors.	-1.63 (0.06)	0.82	0.75	0.89	0.91
36	*The noise of animals gets on my nerves.*	-1.67 (0.06)	1.20	1.17	1.11	1.24
37	I enjoy trips to the countryside.	-1.69 (0.06)	1.14	1.10	1.02	1.15

Note. Items in *italics* were negatively formulated and reverse-coded before statistical analyses. Items with letters are from Bogner and Wiseman (2006). The other items are from Brügger et al. (2011). Item difficulties (δ) and standard errors of measurement (SEM) are expressed in logits. Mean square (*MS*) values—weighted ($_w$) by the item variance—reflect the relative discrepancy between the model's predicted responses and the actual responses used to assess item fit. Whereas the first column of fit statistics represents the scale without considering age-group-biased items (all), the other three columns show MS_w values for 11-13-year-olds, 14-15-year-olds, and 16-18-year-olds. The research was conducted in German. Here, we present the English translations of the items. A 5-point Likert scale was used with Items A-D and Item 37. A 5-point frequency format was used with Items 1, 2, 5-7, 9-11, 16, 18, 19, 23, and 25-27. All other items were presented in a yes-no format.
